# Fetal and Childhood Exposure to Phthalate Diesters and Cognitive Function in Children Up to 12 Years of Age: Taiwanese Maternal and Infant Cohort Study

**DOI:** 10.1371/journal.pone.0131910

**Published:** 2015-06-29

**Authors:** Han-Bin Huang, Hsin-Yi Chen, Pen-Hua Su, Po-Chin Huang, Chien-Wen Sun, Chien-Jen Wang, Hsiao-Yen Chen, Chao A. Hsiung, Shu-Li Wang

**Affiliations:** 1 School of Public Health, National Defense Medical Center, Taipei, Taiwan; 2 Department of Special Education, National Taiwan Normal University, Taipei, Taiwan; 3 Department of Pediatrics, Chung Shan Medical University Hospital, Taichung, Taiwan; 4 National Environmental Health Research Center, National Health Research Institutes, Miaoli, Taiwan; 5 Division of Environmental Health and Occupational Medicine, National Health Research Institutes, Miaoli, Taiwan; 6 Institute of Population Health Sciences, National Health Research Institutes, Zhunan, Taiwan; 7 Department of Public Health, China Medical University, Taichung, Taiwan; INIA, SPAIN

## Abstract

Few studies have examined the association between environmental phthalate exposure and children’s neurocognitive development. This longitudinal study examined cognitive function in relation to pre-and postnatal phthalate exposure in children 2–12 years old. We recruited 430 pregnant women in their third trimester in Taichung, Taiwan from 2001–2002. A total of 110, 79, 76, and 73 children were followed up at ages 2, 5, 8, and 11, respectively. We evaluated the children’s cognitive function at four different time points using the Bayley and Wechsler tests for assessing neurocognitive functions and intelligence (IQ). Urine samples were collected from mothers during pregnancy and from children at each follow-up visit. They were analyzed for seven metabolite concentrations of widely used phthalate esters. These esters included monomethyl phthalate, monoethyl phthalate, mono-butyl phthalate, mono-benzyl phthalate, and three metabolites of di(2-ethylhexyl) phthalate, namely, mono-2-ethylhexyl phthalate, mono(2-ethyl-5-hydroxyhexyl) phthalate, and mono(2-ethyl-5-oxohexyl) phthalate. We constructed a linear mixed model to examine the relationships between the phthalate metabolite concentrations and the Bayley and IQ scores. We found significant inverse associations between the children’s levels of urinary mono(2-ethyl-5-oxohexyl) phthalate and the sum of the three metabolites of di(2-ethylhexyl) phthalate and their IQ scores (β = -1.818; 95% CI: -3.061, -0.574, p = 0.004 for mono(2-ethyl-5-oxohexyl) phthalate; β = -1.575; 95% CI: -3.037, -0.113, p = 0.035 for the sum of the three metabolites) after controlling for maternal phthalate levels and potential confounders. We did not observe significant associations between maternal phthalate exposure and the children’s IQ scores. Children’s but not prenatal phthalate exposure was associated with decreased cognitive development in the young children. Large-scale prospective cohort studies are needed to confirm these findings in the future.

## Introduction

Phthalate esters are a family of industrial chemicals that are widely used as plasticizers or softeners in a variety of commercial products including food packaging, medical equipment, toys, furniture, and cosmetics [1]. Phthalates esters are rapidly metabolized to monoesters and are further oxidized to oxidative metabolites by humans. Urinary phthalate metabolites are broadly used as biomarkers of phthalate exposure in humans [1,2]. In addition, phthalate esters are also considered endocrine disruptors that show antiandrogenic, estrogenic, and antithyroid activities [3].

The developing human brain is uniquely vulnerable to toxic chemical exposures including endocrine disruptors [4]. The major windows of developmental vulnerability occur in utero, during infancy, and in early childhood [5]. As a result of the widespread use of phthalate esters and our subsequent exposure to them, their adverse effects on children’s neurocognitive development have become a significant public health concern [6,7]. To date, only a limited number of epidemiological studies have been published evaluating phthalate exposure and children’s neurocognitive development. In prospective studies, prenatal phthalate exposures have been inversely associated with children’s scores on the Bayley scale [8,9]. One cross-sectional study reported associations between the postnatal phthalate levels of school-age children and their reduced intelligence quotient (IQ) [10]. Experimental research has shown adverse effects of exposure to di(2-ethylhexyl) phthalate (DEHP) and di-*n*-butyl phthalate (DnBP) on pup learning, memory, and healthy brain development [11–13]. Only one study has explored the relationship between prenatal and postnatal phthalate exposure and children’s cognitive function using a prospective follow-up approach [14]. For this reason, we conducted a longitudinal study to evaluate this relationship between prenatal and postnatal exposure to phthalates and cognitive function in children.

## Materials and Methods

### Subjects

The subjects were from a longitudinal birth cohort study of environmental exposures and health in pregnant women and children in central Taiwan, which was a pilot study of the nationwide Taiwan Maternal and Infant Cohort Study. We invited all pregnant women at a local medical center in central Taiwan to join the study between December 1, 2000 and November 30, 2001. The women were between the ages of 25 and 35 years, had a single pregnancy and had no known complications, cigarette smoking, or alcohol consumption during their pregnancies. A total of 430 pregnant women were initially recruited in their third trimester [15]. Only mothers and their children at 2–3 years of age who finished a neurocognitive development assessment (n = 110) were included in the present study and followed up during the study. From the recruited mothers, we followed 110, 79, 76, and 73 children at the ages of 2–3, 5–6, 8–9, and 11–12 years, respectively, from 2003 to 2012. Each mother-child pair participated in three follow-up visits on average and at least two visits including the first follow-up. Thus each child must be studied at both birth and 1^st^ follow-up visit at 2 years of age, and at least once at 5, 8, or 11 year follow-up. At the baseline, the pregnant women answered detailed questionnaires in the obstetrics clinic, including their age, parity, education, medical history, cigarette smoking, alcohol use before and after pregnancy, and home pesticide use. Physicians examined the general physical parameters of the newborns, including gestational age, gender, birth weight and height, head and chest circumferences, and Apgar scores. Every three years, we collected urine specimens from children, estimated scores for the Home Observation for Measurement of the Environment Inventory (HOME) [16], and assessed the children’s neurocognitive development.

### Ethics Statement

This protocol was approved by the Institutional Review Board of the National Health Research Institutes in Taiwan. Prior to the study enrollment, written informed consent was obtained from all participating mothers, who also provided written informed consent on behalf of their children.

### Urinary Phthalate Metabolites

The metabolites of di-methyl phthalate (DMP), di-ethyl phthalate (DEP), di-n-butyl phthalate (DnBP), butyl benzyl phthalate (BBzP), and di(2-ethylhexyl) phthalate (DEHP) measured in this study included monomethyl phthalate (MMP), monoethyl phthalate (MEP), mono-butyl phthalate(MBP),mono-benzyl phthalate(MBzP), mono-2-ethylhexyl phthalate (MEHP), mono(2-ethyl-5-hydroxyhexyl) phthalate (MEHHP), mono(2-ethyl-5-oxohexyl) phthalate (MEOHP), and the sum of the DEHP metabolites (ΣMEHP = MEHP + MEHHP + MEOHP).

Maternal urine was collected from the pregnant women during the third trimester at 28–36 weeks. Phthalate metabolites in the maternal urine were measured at National Cheng Kung University from 2002–2003 as described elsewhere [17]. The detection limits of MMP, MEP, MBP, MBzP, MEHP, MEHHP, and MEOHP were 3.4, 2.2, 1.6, 0.99, 0.55, 0.23, and 0.26 ng mL^-1^, respectively. Children’s urine samples were measured at the National Health Research Institute in 2004 and onwards. Additionally, analysis of spot urine samples from children aged 2, 5, 8, and 11 years was done using the method of Koch et al, with modifications [18,19]. Briefly, the urine samples were incubated at 37°C for 15 minutes. Aliquots of 0.1 ml were transferred to 2.0 ml glass screw-cap vials containing ammonium acetate (20 μl, 1 M, pH 6.5), β-glucuronidase (10 μl), and a mixture of isotopic (^13^C_4_) phthalate metabolite standards (100 μl). All urine samples were incubated at 37°C with the enzyme for 1.5 hours to ensure deconjugation. After hydrolysis, each sample was injected with 270 μl of solvent (5% acetonitrile [ACN] + 0.1% formic acid [FA]) into the glass screw-cap vial and mixed well. The analysis was performed using a quantitative liquid chromatography/electrospray ionization tandem mass spectrometry (LC–ESI-MS/MS) system. The detection limits of MMP, MEP, MBP, MBzP, MEHP, MEHHP, and MEOHP were 0.3, 0.3, 1, 0.3, 0.7, 0.1, and 0.1 ng mL^-1^, respectively. Each subject’s urinary phthalate metabolite concentrations were corrected according to urine creatinine levels. Values below the limit of detection (LOD) were set to LOD/2. The percent of urinary phthalate metabolites above the LOD for pregnant women ranged from 84% to 100%. For the children’s urinary samples, the percent of phthalate metabolites above the LOD at 2–3, 5–6, 8–9, and 11–12 years were 90%–100%, 95%–100%, 99%–100%, and 81%–100%, respectively (S1 Table).

### Assessment of Neurocognitive Development

All of the intellectual evaluations were administered to the children using a standardized protocol by qualified psychologists or well-trained researchers with sufficient validation. At ages 2, 5, 8, and 11 years, the children’s intelligence was assessed using the Bayley Scales of Infant Development-II (BSID-II) [20], the Wechsler Preschool and Primary Scale of Intelligence-Revised (WPPSI-R) [21], the Wechsler Intelligence Scale for Children-Version III (WISC-III) [22], and the Wechsler Intelligence Scale for Children-Fourth Edition (WISC-IV) [23], respectively. The measurement procedure is described in detail in a previous study [24]. In brief, we administered the BSID-II, which is the most widely used measure of infant neurocognitive development. The Mental Development Index (MDI) of the BSID-II is statistically analogous to an IQ score. It includes measurements of acquisition of object constancy, memory learning and problem solving, sensory/perceptual activity, discrimination and response, vocalization and beginning of verbal communication, basis of abstract thinking, complex language, habituation, mental mapping, and mathematical concept formation. We used the Chinese version of WPPSI-R, validated by Chinese standardization and norms in Taiwan [25]. The WPPSI-R has five subsets of verbal skills (arithmetic, comprehension, information, similarities, and vocabulary) and five subsets of visual-spatial skill (block design, geometric design, mazes, object assembly, and picture completion). Additionally, it generates a full-scale IQ. Similarly, the WISC-III also has five subsets of verbal skill, five subsets of performance, and provides a full-scale IQ. The WISC-IV has four different components including four subsets of verbal comprehension index (similarities, vocabulary, comprehension, and information), four subsets of perceptual reasoning index (block design, picture concepts, matrix reasoning, and picture completion), three subsets of working memory index (digit span, letter-number sequence, and arithmetic), and three subsets of processing speed index (coding, symbol search, and cancellation). The WISC-IV also provides a full-scale IQ. A total of 10 certified psychologists who were unaware of the results of the phthalate measurements administered all tests. One senior psychologist trained all testers, randomly reviewed selected forms of each assessment procedure, and checked all completed evaluation forms. Only those validated test scores were included in the final analysis.

### Questionnaire and Covariates

We collected information about the children’s gender, birth weight, children’s age, breastfeeding status, gestational age, and children’s BMI. Additionally, other factors that might confound the relationship between the prenatal and postnatal phthalate exposure and the childrenʼs cognitive development during the study interview were collected. At the baseline, the administered questionnaire was used to obtain demographic data on the pregnant women. These data included maternal age, maternal educational level, parity, cigarette smoking, alcohol use before and after pregnancy, and home pesticide use. A parent also completed the Home Observation for Measurement of the Environment Inventory (HOME) [16] at the four different time points in the clinic. We used the HOME score to evaluate the quality and quantity of cognitive and emotional stimulation in the home environment for each child. The Chinese version of the HOME presented moderate to good reliability in Taiwan [26,27]. To select covariates for inclusion in the multivariate models, the key covariates based on the literature [8,10,24] and Akaike’s Information Criterion (AIC) and log-likelihood ratio test were selected. The covariates included the children’s gender, age, HOME score, birth weight, maternal education, and lactation.

### Statistical Analysis

We used Student *t*-tests for examining the continuous variables while the χ^2^ test was used for the categorical variables. Because the distributions of urinary phthalates were skewed in the samples, we used natural log-transformed values (ln) in the analysis. The associations between the prenatal and postnatal urinary phthalate concentrations and the full-scale IQ scores were assessed using a mixed-model repeat measures analysis after adjusting for fixed covariates (such as age, gender, HOME score, birth weight, maternal education, and lactation). These models treated the participants as random effects, and the first-order autoregressive (AR-1) and variance components were constructed as covariance structures. We also utilized the quartile of urinary phthalate among all the participants to test the non-linear relationship between phthalate exposure and the IQ scores in the same models. Residual and influence analyses were conducted. In addition, we explored the relations between phthalate exposure and the IQ scores in the same models stratified by gender; the interaction effects of gender and phthalate metabolites on IQ scores were not significant (data not shown). A two-sided *P*-value of less than 0.05 was considered statistically significant. All statistical analyses were performed using SAS (version 9.1.3; SAS Institute Inc., Cary, NC).

## Results

### Characteristics of subjects

There were no differences in maternal age, BMI, menarche, maternal educational level, paternal educational level, or birth weight between the participants and participants lost to follow-up, except that the gestational age was slightly older among the study participants (Table 1). The distributions of other variables, including the children’ gender, parity, maternal active/passive cigarette smoke exposure, alcohol intake, and pesticide use at home did not differ between the study subjects and those lost to follow-up.

**Table 1:** Demographic characteristics of the study population.

	Study subjects (*n* = 110)			Subjects lost to follow-up (*n* = 320) ^b^			*P*-value^a^
Continuous variables	Mean	SD	*n*	Mean	SD	*n*	
Maternal age (yr)	29.23	3.99	110	28.56	4.55	285	0.172
Maternal BMI (kg/m^2^)	20.92	3.13	108	20.76	3.16	278	0.646
Menarche (yr)	13.56	1.33	108	13.69	1.32	279	0.364
Birth weight (g)	3170.23	416.45	107	3060.95	483.39	260	0.041
Gestational age (wk)	39.30	1.33	110	38.84	1.71	254	0.014
Maternal educational level (yr)	13.78	1.91	109	13.47	2.17	295	0.187
Paternal educational level (yr)	13.93	2.42	109	13.62	2.34	285	0.815
**Categorical variables**		***n***	**%**		***n***	**%**	
Gender							1.000
Male		58	52.7		124	47.1	
Female		52	42.3		139	52.9	
Before pregnancy: Active smoker							0.765
Yes		7	6.5		23	8.0	
No		101	93.5		264	92.0	
Before pregnancy: Passive smoke							0.677
Yes		47	43.5		133	46.5	
No		61	56.5		153	53.5	
During pregnancy: Active smoker							0.453
Yes		1	0.9		7	2.4	
No		109	99.1		279	97.6	
Alcohol consumption							0.529
Yes		2	1.8		11	3.8	
No		108	98.2		275	96.2	
Pesticide use at home							0.669
Yes		33	30.0		192	67.1	
No		77	70.0		94	32.9	
Parity							0.101
1^st^		109	99.1		185	95.4	
2^nd^		1	0.9		9	4.6	
Lactation							0.548
Yes		96	92.3		232	89.6	
No		8	7.7		27	10.4	

^a^Statistical methods: Independent *t*-test and χ^2^ test, as appropriate.

^b^ A total of 320 subjects with complete data at baseline were lost to follow-up.

We found that the concentrations of phthalate metabolites in children, including MMP, MEP, MBP, MBzP, MEHP, MEHHP, MEOHP, and ΣMEHP, were significantly related to age (Table 2). Levels of urinary phthalate metabolites in the children at 2 years old were higher than that of children at 11 years old. Generally, the levels of MMP, MEP, MBP, and ΣMEHP metabolites decreased as the children’s ages increased. For the trend of MBzP, the levels increase from 2–3 years to 5–6 years and then decrease from 5–6 years to 11–12 years. Furthermore, no significant correlations between the same phthalate metabolite at different ages were found, except for MBP at ages 2–3 and 5–6, ΣMEHP at ages 2–3 and 8–9, and ΣMEHP at ages 8–9 and 11–12, which were significantly correlated (S2 Table).

**Table 2:** Concentrations (geometric mean, GM) of maternal and childrenʼs urinary phthalates (μg/g creatinine), HOME scores, and intelligence quotients (IQs) at the four follow-up points.

	Pregnant women		1^st^ visit (2–3 years)		2^nd^ visit (5–6 years)		3^rd^ visit (8–9 years)		4^th^ visit (11–12 years)		*P* for trend^b^
Variables	GM(95% CI)	*n* ^*a*^	GM(95% CI)	*n*	GM(95% CI)	*n*	GM(95% CI)	*n*	GM(95% CI)	*n*	
MMP	49.84(40.92, 60.71)	100	14.58(12.16, 17.49)	93	12.34(9.73, 15.64)	74	6.64(5.19, 8.50)	75	8.60(6.12, 12.09)	73	<0.001
MEP	66.61(55.73, 79.61)	100	34.35(26.78, 44.06)	93	16.18(12.82, 20.43)	74	13.67(10.75, 17.40)	75	7.63(4.99, 11.67)	73	<0.001
MBP	77.87(64.84, 93.52)	100	170.12(145.19, 199.33)	93	111.65(96.50, 129.18)	74	83.68(69.70, 100.48)	75	74.86(65.64, 85.37)	73	<0.001
MBzP	17.43(15.15, 20.05)	100	7.45(5.90, 9.42)	93	14.82(12.10, 18.16)	74	10.16(8.00, 12.91)	75	3.21(2.50, 4.10)	73	<0.001
MEHP	19.79(16.38, 23.92)	100	16.26(13.67, 19.35)	93	13.31(10.30, 17.20)	74	8.34(6.28, 11.07)	75	10.07(8.14, 12.44)	73	<0.001
MEHHP	8.49(5.97, 12.09)	100	93.38(78.78, 110.68)	93	91.30(72.43, 115.08)	74	42.10(33.56, 52.80)	75	33.16(28.98, 37.95)	73	<0.001
MEOHP	12.97(9.23, 18.21)	100	65.83(54.68, 79.26)	93	52.51(43.14, 63.93)	74	37.07(29.69, 46.28)	75	24.29(19.38, 30.44)	73	<0.001
ΣMEHP^c^	58.69(48.32, 71.30)	100	184.55(158.14, 215.37)	93	167.61(136.77, 205.39)	74	89.46(71.38, 112.12)	75	72.11(63.08, 82.44)	73	<0.001
HOME score (mean ± SD)	-	-	40.30 ± 4.01	107	45.59 ± 5.10	79	46.01 ± 6.13	75	44.88 ± 8.23	69	<0.001
IQ^d^ (mean ± SD)	-	-	95.37 ± 12.41	110	105.93 ± 13.66	76	109.41 ± 11.76	76	109.15 ± 13.21	72	<0.001

^a^Ten pregnant women could not provide sufficient urine samples; the total numbers of pregnant women were 100 subjects.

^b^Mixed model was used to test for age trend of childrenʼs urinary phthalate levels, HOME score, and IQ.

^c^ΣMEHP = MEHP + MEHHP + MEOHP.

^d^IQ: The mental development index scores of the Bayley Scales were used to assess the IQ of children aged 2–3 years. The Wechsler Scales were to evaluate the IQ of children aged 5–12 years.

### Relationship between phthalate metabolites and IQ

Table 3 presented the association between IQ score and maternal and children’s urinary phthalate metabolite levels using a linear mixed model adjusted for age, gender, HOME score, birth weight, lactation, and maternal education. No maternal phthalate metabolite levels were significantly associated with the children’s IQ scores. MEOHP and ΣMEHP were significantly inversely associated with IQ scores (MEOHP, β = -1.818, 95% CI: -3.061, -0.574, p = 0.004; ΣMEHP, β = -1.575, 95% CI: -3.037, -0.113, p = 0.035). A one-fold incremental increase in the MEOHP and ΣMEHP altered the IQ scores by -1.26 and -1.09, respectively. The children’s other phthalate metabolites, including MMP, MEP, MBP, MBzP, MEHP, and MEHHP, were inversely associated with the IQ scores but were not statistically significant. We also examined the effects of prenatal and postnatal exposure to phthalate metabolites on IQ scores at different ages among the children (S3 Table), and the results were consistent with those in Table 3. Additionally, our findings shown in Table 3 were similar to those with adjustment for creatinine levels as the covariates in the regression model (S4 Table).

**Table 3:** Associations of intelligence quotient (IQ) scores with mothers’ and childrenʼs urinary phthalate concentrations by linear mixed model (n^a^ = 251).

Phthalate metabolite (μg/g creatinine)	Beta IQ	95% CI	*P*-value
Model 1^b^ ^,^ ^c^			
Ln child MMP	-1.043	-2.164, 0.076	0.068
Ln maternal MMP	-0.255	-2.418, 1.909	0.817
Model 2^b^ ^,^ ^c^			
Ln child MEP	-0.579	-1.515, 0.356	0.224
Ln maternal MEP	1.593	-0.695, 3.881	0.171
Model 3^b^ ^,^ ^c^			
Ln child MBP	-1.684	-3.496, 0.128	0.068
Ln maternal MBP	-0.215	-2.496, 2.067	0.853
Model 4^b^ ^,^ ^c^			
Ln child MBzP	-0.934	-2.118, 0.250	0.121
Ln maternal MBzP	-0.056	-3.097, 2.985	0.971
Model 5^b^ ^,^ ^c^			
Ln child MEHP	-1.026	-2.184, 0.133	0.082
Ln maternal MEHP	-1.069	-3.259, 1.122	0.337
Model 6^b^ ^,^ ^c^			
Ln child MEHHP	-1.216	-2.601, 0.170	0.085
Ln maternal MEHHP	-0.289	-1.459, 0.882	0.627
Model 7^b^ ^,^ ^c^			
**Ln child MEOHP**	**-1.818**	**-3.061, -0.574**	**0.004**
Ln maternal MEOHP	0.264	-0.928, 1.457	0.662
Model 8^b^ ^,^ ^c^			
**Ln child ΣMEHP** ^**d**^	**-1.575**	**-3.037, -0.113**	**0.035**
Ln maternal ΣMEHP^**d**^	-0.119	-2.197, 1.959	0.910

^a^The number of observations (n) represents the sum of all subjects studied at both birth and 1^st^ follow-up visit at 2 years of age, and at least once at 5, 8, or 11 year follow-up.

^b^Adjusted for gender, HOME score, birth weight, maternal education, lactation, and children’s age.

^c^Maternal and children’s levels of urinary phthalate were both independent variables to predict IQ scores in the model.

^d^ΣMEHP = MEHP + MEHHP + MEOHP.

**Fig 1:**
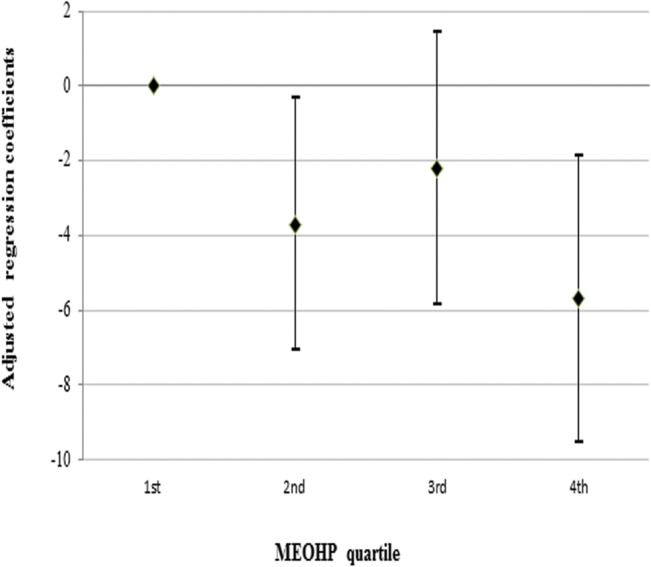
Adjusted regression coefficients (β [95% CI]) for change in children’s cognitive development assessed by Bayley and Wechsler IQ scores in relation to 2, 5, 8, 11 year old children’s urinary MEOHP quartile. Values were calculated using a linear mixed model adjusting for age, gender, HOME score, birth weight, maternal education, lactation, and maternal phthalate metabolite levels.

To test the nonlinear relationship between phthalate metabolites and the IQ scores, we plotted the IQ scores by the quartiles of urinary phthalate metabolite levels in the children. Mean IQ scores were 5.69 points lower (95% CI: -9.52, -1.87) and 3.69 points lower (95% CI: -7.06, -0.32) in the fourth and second quartiles for MEOHP compared to the first quartile, respectively (Fig 1). Similar results were observed including MBP, MEHHP, and ΣMEHP (S1 Fig).

## Discussion

The objective of our study was to examine the associations between prenatal and postnatal phthalate exposure and neurocognitive development among children. We found an inverse relationship between postnatal exposure to MEOHP and ΣMEHP and the children’s IQ scores after adjustment for covariates. Our results indicated that long-term and persistent exposure to phthalates caused adverse effects on cognitive development in children. Consistent with our study, another showed inverse associations between postnatal exposure to MEHP, MEOHP, and the sum of secondary metabolites for DEHP and the IQ scores among school-age children in a cross-sectional study [10]. The estimated effect sizes (β = -2.2, 95% CI: -3.6,-0.8 for MEOHP) reported by the previous study [10] were similar to those (β = -1.7, 95% CI: -3.0, -0.5 for MEOHP) of our model without adjusting for maternal phthalate metabolite levels. In terms of prenatal phthalate exposure, no significant associations between maternal urinary phthalate metabolites and the children’s IQ scores were observed in our study. Similarly, two other studies also found that maternal urinary DEHP metabolites were not associated with children’s Mental Development Index scores at 2–3 years of age [9,28]. However, another study in Korea found that prenatal exposure to DEHP metabolites was negatively associated with the Mental Development Index scores of infants at 6 months [8]. The inconsistency with other studies [8,14] could be due to the differences in the children’s age, exposure profiles, sample size, ethnic and social groups, and adjusted covariates.

The mechanism underlying the adverse effects of phthalates on neurocognitive development is uncertain. Cognitive function is associated with the regulation of the neurotransmitter system. Low-dose phthalates may decrease the number of midbrain dopaminergic neurons, tyrosine hydroxylase biosynthetic activity [13], and tyrosine hydroxylase immunoreactivity [29]. In addition, several studies have reported the possible antagonistic effects of phthalates on thyroid function in both in vitro and in vivo studies [30,31]. Thyroid hormones play a fundamental role in neurocognitive development and hippocampal function; delayed, or impaired brain differentiation, and hippocampal dysfunction often lead to deficits in learning and memory in rats [32,33]. Exposure to phthalates may be associated with altered thyroid activity in both pregnant women [34] and children [35]. Previous studies have reported that neonatal hypothyroidism and subclinical hypothyroidism could affect children’s cognition [36–38].

An alternative biological explanation is that phthalates activate the peroxisome proliferator-activated receptors (PPARs) [39]. These receptors are found in developing neural tubes [40]. Some studies have observed that ligands of PPAR played roles in lipid metabolism, cellular proliferation, and the inflammatory response [41]. Its signal transduction pathway is involved in the progression of neurodegenerative and psychiatric diseases and its relation to cognitive function [42].

We also found that phthalate metabolite concentrations were inversely associated with age, which is in agreement with previous studies [43,44]. The children showed higher urinary phthalate metabolite concentrations than adults [43]. In addition, our data showed negative associations between children’s phthalate metabolites levels and IQ scores at different ages. These results support an assumption that early life phthalate exposure plays a significant role in children’s cognitive development.

With regard to long-term trend in phthalate exposures, the levels of MBP, MBzP, andΣMEHP metabolites in children’s urine from the study by Langer et al. [43] have shown decreasing trends, which were in agreement with our findings. However, MEP levels remained about the same during the last decade in this Danish study, which was different from our findings. As a whole, our subjects had higher levels of MMP, MBP, andΣMEHP metabolites compared to children in the United States and Germany [43–45]. In contrast, levels of MEP and MBzP were much lower than in children in the United States and Germany. Levels of exposure in pregnancy in our study compared with previous studies [45,46] showed analogous findings. Because MBzP levels in the children in the present study were close to levels measured in a previous Taiwanese study [47], we suggest BBzP levels in Taiwan are consistently lower. A different lifestyle, dietary habits, different exposure routes, durations, concentrations, and different rate of metabolism may explain these inconsistencies [48,49].

There are some limitations to this study. First, we found significant inverse associations between postnatal MEOHP exposure and IQ scores in boys; however, the sample size was insufficient to detect the statistical significance in relation to the interaction effects of gender and phthalate metabolites on IQ scores. Previous studies have shown no consistent or apparent gender differences regarding phthalate exposure and neurocognitive development [9,28]. Second, spot urine was only collected from the pregnant women in the third trimester. Because of the short half-lives of phthalates and the episodic nature of exposure, single spot urine measurements might not reflect long-term exposure among pregnant women. However, previous studies have reported that phthalates detected in spot urine samples from pregnant women in the third trimester indicated moderate reliability for the presence of phthalates for a period ranging from weeks to months [8,50–52]. Third, we did not collect maternal IQ data at the recruitment time for consideration of adjustment. Considering maternal IQ as confounding variable in the case of lead toxicity, we might want to adjust for maternal IQ in future studies [53]. However, adjusting for a confounder that has a stronger effect than the variable of interest can result in underestimating the actual effect of the variable. In addition, we obtained information on the potential covariates related to maternal IQ, including socioeconomic status and the HOME score. Consequently, our results could indicate minimal effects from the maternal IQ.

## Conclusions

Higher postnatal urinary phthalate metabolite levels were associated with lower IQ scores in children 2–12 years of age, suggesting that continuing exposure to environmental phthalates could adversely affect children’s cognitive development. However, fetal exposure to phthalate was not significantly and independently associated with decrements in IQ scores. Large-scale prospective cohort studies are needed to confirm these findings in the future.

## Supporting Information

S1 Fig Adjusted regression coefficients [β (95% CI)] for change in children’s cognitive development assessed by Bayley and Wechsler IQ scores in relation to 2, 5, 8, 11 year old children’s urinary quartile of MMP (A), MEP (B), MBP (C), MBzP (D), MEHP (E), MEHHP (F), and ΣMEHP (G). Values were calculated using a linear mixed model adjusting for age, gender, HOME score, birth weight, maternal education, lactation, and maternal phthalate metabolite levels.
(PDF)

S1 Table The percentage of phthalate metabolites above limit of detection (LOD) in maternal and children’s urine. (DOCX)

S2 Table Spearman’s rho correlation between pregnant women’s and children’s urinary phthalate metabolite levels at 2–3, 5–6, 8–9, and 11–12 years of age. (DOCX)

S3 Table Multiple regression analysis of maternal and children’s urinary phthalate metabolite levels and intelligence quotient (IQ)^d^ scores at 2–3, 5–6, 8–9, and 11–12 years of age. (DOCX)

S4 Table Associations between intelligence quotient (IQ) scores and mothers’ and childrenʼs urinary phthalate concentrations by linear mixed model (n ^a^ = 251). (DOCX)

S5 Table Associations between intelligence quotient (IQ) scores and mothers’ and childrenʼs urinary phthalate concentrations by linear mixed model (n ^a^ = 196), with excluding mother’s and children’s urinary creatinine below 2 g/L. (DOCX)
