# Supplementary material for: Fetal and Childhood Exposure to Phthalate Diesters and Cognitive Function in Children Up to 12 Years of Age: Taiwanese Maternal and Infant Cohort Study
Source: PLoS One. 2015 Jun 29;10(6):e0131910. doi: 10.1371/journal.pone.0131910 (PMC4488303; doi:10.1371/journal.pone.0131910)
Supplement: S1 Fig — Values were calculated using a linear mixed model adjusting for age, gender, HOME score, birth weight, maternal education, lactation, and maternal phthalate metabolite levels. (PDF) [file pone.0131910.s001.pdf]

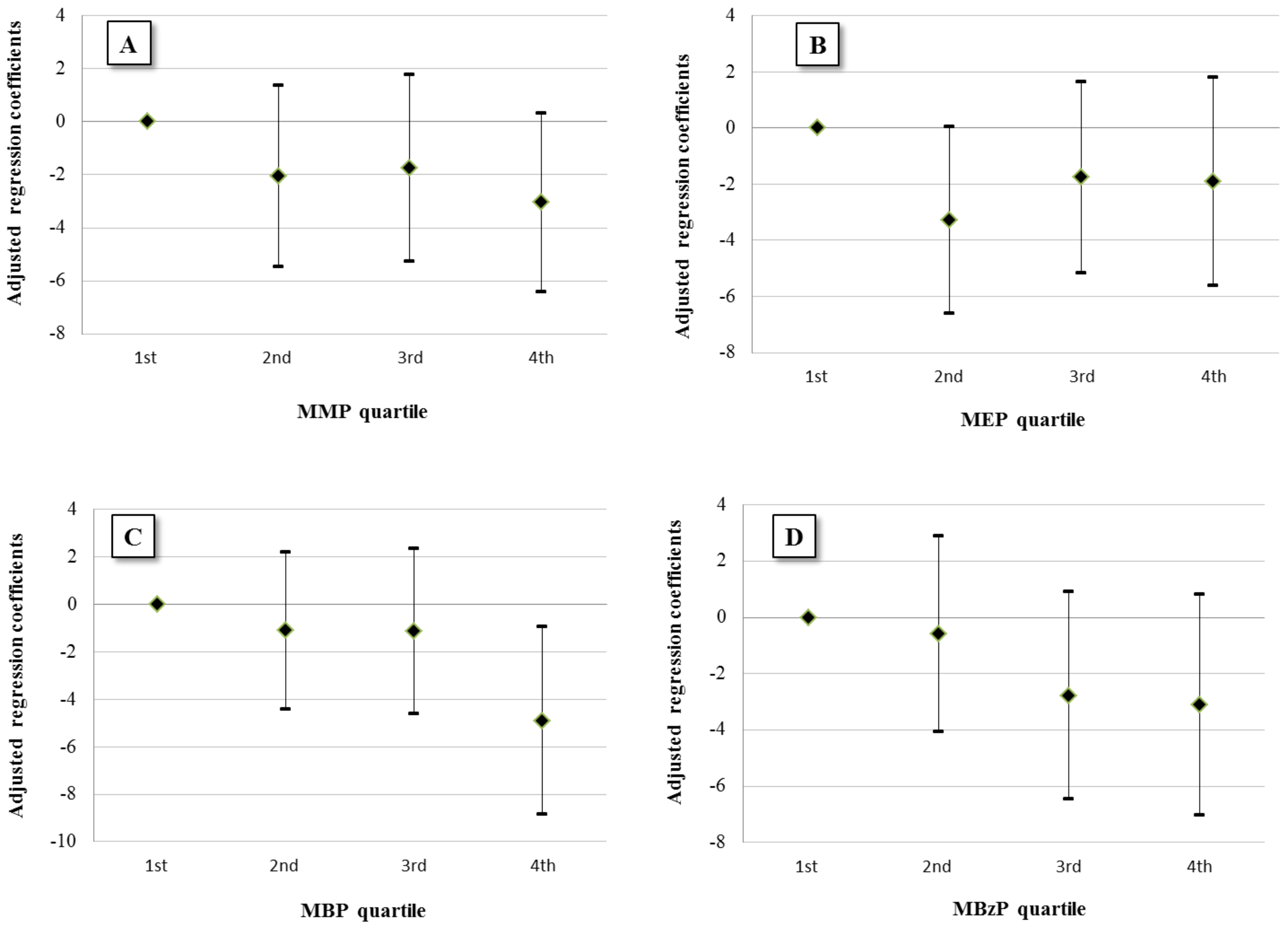
A
Adjusted regression coefficients
4
2
0
-2
-4
-6
-8
1st
2nd
3rd
4th
MMP quartile
B
Adjusted regression coefficients
4
2
0
-2
-4
-6
-8
1st
2nd
3rd
4th
MEP quartile
C
Adjusted regression coefficients
4
2
0
-2
-4
-6
-8
-10
1st
2nd
3rd
4th
MBP quartile
D
Adjusted regression coefficients
4
2
0
-2
-4
-6
-8
1st
2nd
3rd
4th
MBzP quartile

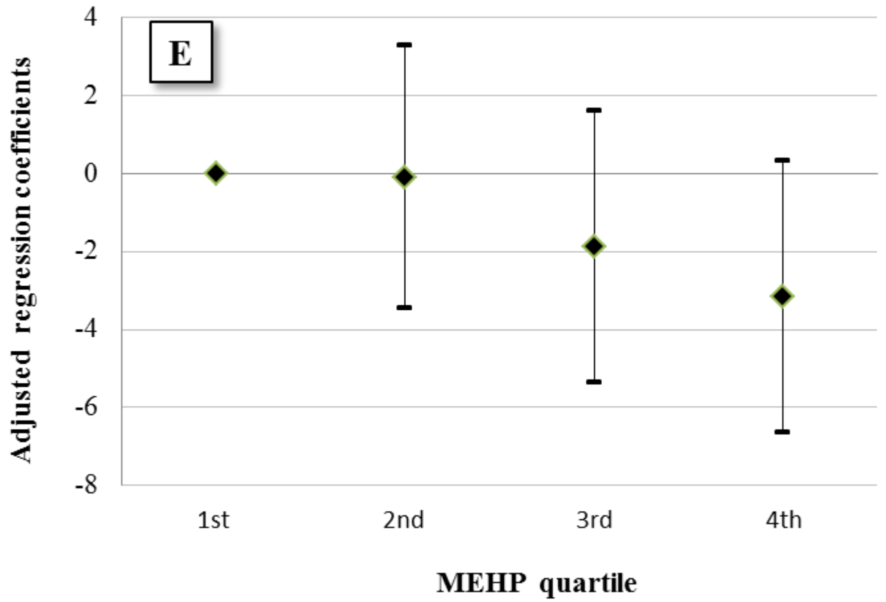
E
Adjusted regression coefficients
4
2
0
-2
-4
-6
-8
1st
2nd
3rd
4th
MEHP quartile

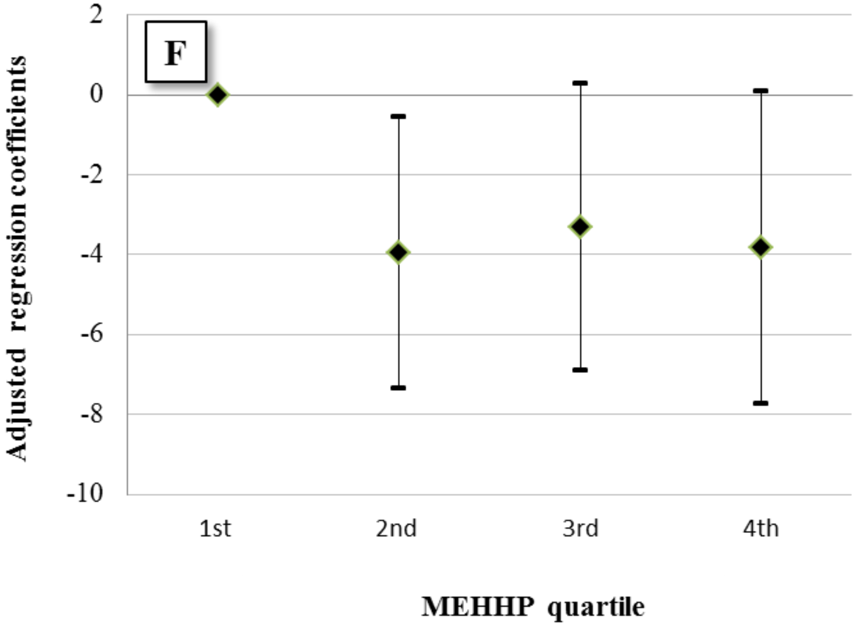
F
Adjusted regression coefficients
2
0
-2
-4
-6
-8
-10
1st
2nd
3rd
4th
MEHHP quartile

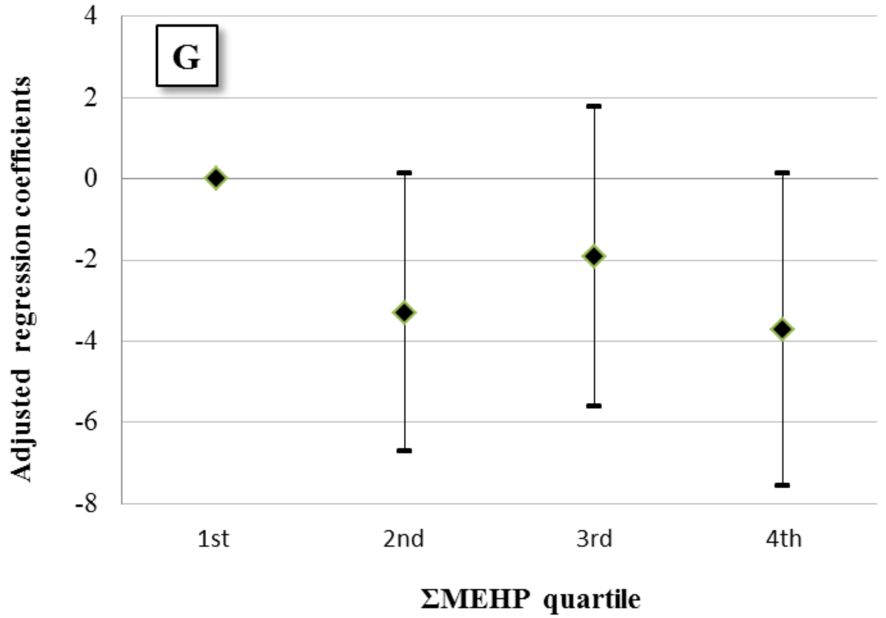
G
Adjusted regression coefficients
4
2
0
-2
-4
-6
-8
1st
2nd
3rd
4th
ΣMEHP quartile

S1 Fig. Adjusted regression coefficients [β(95% CI)] for change in children's cognitive development by Bayley and Wechsler IQ scores in relation to 2, 5, 8, 11 year old children's urinary quartile of MMP (A), MEP (B), MBP (C), MBzP (D), MEHP (E), MEHHP (F), and ΣMEHP (G). Values were shown using linear mixed model adjusting for age, gender, HOME score, birth weight, maternal education, lactation, and maternal phthalate metabolite levels.
