# Supplementary material for: Fetal and Childhood Exposure to Phthalate Diesters and Cognitive Function in Children Up to 12 Years of Age: Taiwanese Maternal and Infant Cohort Study
Source: PLoS One. 2015 Jun 29;10(6):e0131910. doi: 10.1371/journal.pone.0131910 (PMC4488303; doi:10.1371/journal.pone.0131910)
Supplement: S1 Table — (DOCX) [file pone.0131910.s002.docx]

**S1 Table.** The percentage of phthalate metabolites above limit of detection (LOD) in maternal and children’s urine

|  | Pregnant women | | 1^st^ (2–3 years) | | 2^nd^ (5–6 years) | | 3^rd^ (8–9 years) | | 4^th^ (11–12 years) | |
| --- | --- | --- | --- | --- | --- | --- | --- | --- | --- | --- |
| Variables | % >LOD | *n=100* | % >LOD | *n =93* | % >LOD | *n =74* | % >LOD | *n =75* | % >LOD | *n =73* |
| MMP | 100 | 100 | 96.8 | 90 | 100 | 74 | 98.7 | 74 | 87.8 | 64 |
| MEP | 100 | 100 | 98.9 | 92 | 97.3 | 72 | 98.7 | 74 | 80.8 | 59 |
| MBP | 100 | 100 | 100 | 93 | 100 | 74 | 100 | 75 | 100 | 73 |
| MBzP | 100 | 100 | 90.3 | 84 | 98.6 | 73 | 98.7 | 74 | 91.8 | 67 |
| MEHP | 98 | 98 | 98.9 | 92 | 94.6 | 70 | 100 | 75 | 97.3 | 71 |
| MEHHP | 84 | 84 | 100 | 93 | 100 | 74 | 100 | 75 | 100 | 73 |
| MEOHP | 91 | 91 | 98.9 | 92 | 100 | 74 | 100 | 75 | 97.3 | 71 |
