# Supplementary material for: Fetal and Childhood Exposure to Phthalate Diesters and Cognitive Function in Children Up to 12 Years of Age: Taiwanese Maternal and Infant Cohort Study
Source: PLoS One. 2015 Jun 29;10(6):e0131910. doi: 10.1371/journal.pone.0131910 (PMC4488303; doi:10.1371/journal.pone.0131910)
Supplement: S2 Table — (DOCX) [file pone.0131910.s003.docx]

**S2 Table.** Spearman’s rho correlation between pregnant women’s and children’s urinary phthalate metabolite levels at 2–3, 5–6, 8–9, and 11–12 years.

^a^LMM = MMP + MEP

^b^ΣMEHP = MEHP + MEHHP + MEOHP

^＊^*p* < 0.05; ^＊＊^*p* < 0.01

^a^LMM = MMP + MEP

^b^ΣMEHP = MEHP + MEHHP + MEOHP

^＊^*p* < 0.05; ^＊＊^*p* < 0.01

|  | 2–3 years | | | | 5–6 years | | | | 8–9 years | | | | 11–12 years | | | |
| --- | --- | --- | --- | --- | --- | --- | --- | --- | --- | --- | --- | --- | --- | --- | --- | --- |
| Correlation coefficient | LMM^a^ | MBP | MBzP | ΣMEHP^b^ | LMM^a^ | MBP | MBzP | ΣMEHP^b^ | LMM^a^ | MBP | MBzP | ΣMEHP^b^ | LMM^a^ | MBP | MBzP | ΣMEHP^b^ |
| Pregnant women |  |  |  |  |  |  |  |  |  |  |  |  |  |  |  |  |
| LMM^a^ | 0.053 | 0.020 | -0.078 | 0.064 | 0.029 | -0.058 | -0.037 | 0.135 | 0.037 | 0.045 | -0.012 | -0.043 | 0.071 | -0.156 | 0.061 | -0.188 |
| MBP | 0.067 | 0.123 | -0.216 | 0.114 | 0.055 | 0.105 | -0.047 | -0.135 | 0.033 | 0.345^＊＊^ | 0.216 | 0.182 | 0.059 | 0.035 | 0.110 | 0.009 |
| MBzP | -0.002 | -0.050 | -0.285^＊＊^ | 0.092 | 0.023 | -0.043 | -0.390^＊＊^ | -0.050 | 0.022 | 0.115 | 0.082 | 0.079 | 0.033 | 0.046 | 0.127 | -0.039 |
| ΣMEHP^b^ | 0.072 | -0.014 | -0.301^＊＊^ | 0.129 | 0.010 | -0.074 | -0.087 | -0.068 | -0.064 | 0.025 | 0.083 | 0.082 | 0.034 | -0.020 | 0.160 | -0.067 |
| 2–3 years |  |  |  |  |  |  |  |  |  |  |  |  |  |  |  |  |
| LMM^a^ |  |  |  |  | -0.051 | 0.031 | 0.096 | -0.079 | 0.061 | 0.016 | -0.053 | 0.069 | 0.006 | 0.106 | 0.032 | -0.023 |
| MBP |  |  |  |  | 0.188 | 0.367^＊＊^ | 0.256 | 0.074 | -0.184 | -0.046 | -0.098 | -0.008 | -0.005 | 0.004 | -0.034 | -0.003 |
| MBzP |  |  |  |  | 0.039 | 0.128 | 0.180 | -0.038 | -0.058 | -0.125 | -0.091 | -0.245 | -0.012 | -0.105 | -0.095 | -0.021 |
| ΣMEHP^b^ |  |  |  |  | 0.029 | 0.080 | -0.134 | -0.150 | -0.116 | 0.150 | -0.047 | 0.293^＊^ | 0.116 | 0.045 | 0.132 | 0.047 |
| 5–6 years |  |  |  |  |  |  |  |  |  |  |  |  |  |  |  |  |
| LMM^a^ |  |  |  |  |  |  |  |  | 0.111 | 0.116 | 0.073 | -0.120 | -0.020 | -0.028 | -0.091 | -0.141 |
| MBP |  |  |  |  |  |  |  |  | -0.124 | -0.049 | 0.085 | -0.033 | 0.145 | 0.068 | -0.159 | 0.033 |
| MBzP |  |  |  |  |  |  |  |  | -0.228 | -0.229^＊^ | 0.030 | -0.285^＊^ | -0.136 | -0.048 | 0.002 | -0.225 |
| ΣMEHP^b^ |  |  |  |  |  |  |  |  | 0.118 | 0.078 | 0.125 | 0.187 | -0.048 | 0.103 | 0.089 | 0.096 |
| 8–9 years |  |  |  |  |  |  |  |  |  |  |  |  |  |  |  |  |
| LMM^a^ |  |  |  |  |  |  |  |  |  |  |  |  | 0.127 | 0.081 | 0.293^＊^ | 0.108 |
| MBP |  |  |  |  |  |  |  |  |  |  |  |  | 0.088 | 0.196 | 0.341^＊＊^ | 0.245^＊^ |
| MBzP |  |  |  |  |  |  |  |  |  |  |  |  | 0.165 | 0.133 | 0.166 | 0.256^＊^ |
| ΣMEHP^b^ |  |  |  |  |  |  |  |  |  |  |  |  | 0.092 | 0.152 | 0.310^＊^ | 0.314^＊^ |
