# Supplementary material for: Fetal and Childhood Exposure to Phthalate Diesters and Cognitive Function in Children Up to 12 Years of Age: Taiwanese Maternal and Infant Cohort Study
Source: PLoS One. 2015 Jun 29;10(6):e0131910. doi: 10.1371/journal.pone.0131910 (PMC4488303; doi:10.1371/journal.pone.0131910)
Supplement: S3 Table — (DOCX) [file pone.0131910.s004.docx]

**S3 Table.** Multiple regression analysis of maternal and children’s urinary phthalate metabolite levels and intelligence quotient (IQ) d scores at 2–3, 5–6, 8–9, and 11–12 years

|  | 2–3 years (n=76) | | | 5–6 years (n=61) | | | 8–9 years (n=58) | | | 11–12 years (n=56) | | | |
| --- | --- | --- | --- | --- | --- | --- | --- | --- | --- | --- | --- | --- | --- |
| Variables (µg/g creatinine) | Beta | 95% CI | *p*-value | Beta | 95% CI | *p*-value | Beta | 95% CI | *p*-value | | Beta | 95% CI | *p*-value |
| Model 1^a,b^ |  |  |  |  |  |  |  |  |  | |  |  |  |
| Ln MMP | 0.573 | -3.153, 3.299 | 0.676 | -2.290 | -5.377, 0.796 | 0.143 | -0.919 | -4.044, 2.205 | 0.557 | | -1.589 | -4.691, 1.513 | 0.308 |
| Ln maternal urinary MMP | 0.462 | -2.270, 3.194 | 0.737 | 0.919 | -2.342, 4.179 | 0.674 | -0.840 | -4.445, 2.765 | 0.642 | | -2.301 | -5.841, 1.240 | 0.198 |
| Model 2 ^a,b^ |  |  |  |  |  |  |  |  |  | |  |  |  |
| Ln MEP | -0.312 | -2.421, 1.796 | 0.768 | -0.482 | -3.919, 2.955 | 0.780 | -3.191 | -6.257, -0.125 | 0.042 | | 0.035 | -1.797, 1.868 | 0.969 |
| Ln maternal urinary MEP | 1.630 | -1.082, 4.341 | 0.235 | 1.372 | -2.255, 4.998 | 0.451 | 1.831 | -1.317, 4.979 | 0.248 | | 1.579 | -1.965, 5.123 | 0.375 |
| Model 3 ^a,b^ |  |  |  |  |  |  |  |  |  | |  |  |  |
| Ln MBP | -2.845 | -6.133, 0.443 | 0.089 | 4.122 | -2.321, 10.566 | 0.205 | -2.886 | -7.291, 1.518 | 0.194 | | 0.189 | -5.732, 6.110 | 0.949 |
| Ln maternal urinary MBP | 0.913 | -2.021, 3.847 | 0.537 | -0.782 | -4.557, 2.994 | 0.680 | -0.899 | -4.410, 2.612 | 0.609 | | -0.640 | -4.282, 3.003 | 0.726 |
| Model 4 ^a,b^ |  |  |  |  |  |  |  |  |  | |  |  |  |
| Ln MBzP | -1.536 | -3.806, 0.734 | 0.181 | 1.265 | -2.745, 5.276 | 0.530 | -2.885 | -6.048, 0.279 | 0.073 | | -0.414 | -3.879, 3.051 | 0.811 |
| Ln maternal urinary MBzP | 0.237 | -3.839, 3.642 | 0.898 | 0.614 | -4.814, 6.042 | 0.821 | -1.104 | -6.286, 4.078 | 0.671 | | 2.591 | -2.980, 8.162 | 0.354 |
| Model 5 ^a,b^ |  |  |  |  |  |  |  |  |  | |  |  |  |
| Ln MEHP | 1.596 | -1.521, 4.712 | 0.311 | -0.848 | -3.778, 2.082 | 0.564 | -3.141 | -5.369, -0.913 | 0.007 | | -0.889 | -4.900, 3.122 | 0.658 |
| Ln maternal urinary MEHP | 0.261 | -2.351, 2.873 | 0.842 | -1.813 | -5.444, 1.819 | 0.321 | -0.610 | -3.655, 2.434 | 0.689 | | -0.629 | -4.165, 2.906 | 0.722 |
|  |  |  |  |  |  |  |  |  |  | |  |  |  |

**S3 Table. (cont.)**

|  | 2–3 years (n=76) | | | | 5–6 years (n=61) | | | | 8–9 years (n=58) | | | | 11–12 years (n=56) | | |
| --- | --- | --- | --- | --- | --- | --- | --- | --- | --- | --- | --- | --- | --- | --- | --- |
| Variables (µg/g creatinine) | Beta | 95% CI | *p*-value | Beta | | 95% CI | *p*-value | Beta | | 95% CI | *p*-value | Beta | | 95% CI | *p*-value |
| Model 6 ^a,b^ |  |  |  |  | |  |  |  | |  |  |  | |  |  |
| Ln MEHHP | -1.425 | -4.536, 1.685 | 0.364 | 1.957 | | -1.343, 5.256 | 0.240 | -4.182 | | -7.130, -1.234 | 0.006 | -7.510 | | -13.236, -1.785 | 0.011 |
| Ln maternal urinary MEHHP | -0.115 | -1.630, 1.399 | 0.880 | -0.288 | | -2.117, 1.542 | 0.754 | 0.705 | | -0.938, 2.348 | 0.393 | -0.145 | | -1.900, 1.610 | 0.869 |
| Model 7 ^a,b^ |  |  |  |  | |  |  |  | |  |  |  | |  |  |
| Ln MEOHP | -2.882 | -5.550, -0.214 | 0.035 | 1.298 | | -2.483, 5.079 | 0.494 | -4.318 | | -7.263, -1.374 | 0.005 | -3.154 | | -6.229, -0.079 | 0.045 |
| Ln maternal urinary MEOHP | 0.617 | -0.852, 2.085 | 0.405 | 0.787 | | -1.167, 2.741 | 0.423 | 1.406 | | -0.390, 3.201 | 0.122 | -0.425 | | -2.397, 1.547 | 0.667 |
| Model 8 ^a,b^ |  |  |  |  | |  |  |  | |  |  |  | |  |  |
| Ln ΣMEHP^c^ | -2.228 | -5.548, 1.091 | 0.185 | 1.524 | | -2.178, 5.225 | 0.413 | -4.146 | | -7.047, -1.246 | 0.006 | -6.156 | | -11.847, -0.464 | 0.035 |
| Ln maternal urinary ΣMEHP^c^ | 1.266 | -1.305, 3.836 | 0.329 | -0.566 | | -3.753, 2.622 | 0.723 | 1.524 | | -1.458, 4.505 | 0.310 | -0.163 | | -3.189, 2.863 | 0.914 |
|  |  |  |  |  | |  |  |  | |  |  |  | |  |  |

^a^adjusted for gender, HOME score, birth weight, maternal education, and lactation.

^b^Maternal and children’s levels of urinary phthalate were both independent variables to predict IQ scores in the model.

^c^ΣMEHP= MEHP+ MEHHP+MEOHP.

^d^IQ: The mental development index (MDI) scores of the Bayley Scale were to assess IQ for children aged 2–3 years old; the Wechsler Scales were to assess IQ for children aged 5–12 years old.
