# Supplementary material for: Fetal and Childhood Exposure to Phthalate Diesters and Cognitive Function in Children Up to 12 Years of Age: Taiwanese Maternal and Infant Cohort Study
Source: PLoS One. 2015 Jun 29;10(6):e0131910. doi: 10.1371/journal.pone.0131910 (PMC4488303; doi:10.1371/journal.pone.0131910)
Supplement: S4 Table — (DOCX) [file pone.0131910.s005.docx]

**S4 Table.** Associations between intelligence quotient (IQ) scores and mothers’ and childrenʼs urinary phthalate concentrations by linear mixed model (n ^a^=251)

| Variables (ng/ml) | Beta | 95% CI | *p*-value |
| --- | --- | --- | --- |
| Model 1 ^b, c^ |  |  |  |
| Ln MMP | -1.045 | -2.176, 0.087 | 0.070 |
| Ln maternal MMP | 0.031 | -2.336, 2.399 | 0.979 |
| Model 2 ^b, c^ |  |  |  |
| Ln MEP | -0.588 | -1.527, 0.350 | 0.217 |
| Ln maternal MEP | 2.420 | -0.176, 5.016 | 0.068 |
| Model 3 ^b, c^ |  |  |  |
| Ln MBP | -1.762 | -3.618, 0.094 | 0.063 |
| Ln maternal MBP | -0.013 | -2.467, 2.442 | 0.992 |
| Model 4 ^b, c^ |  |  |  |
| Ln MBzP | -0.919 | -2.111, 0.274 | 0.130 |
| Ln maternal MBzP | 0.736 | -3.122, 4.593 | 0.707 |
| Model 5 ^b, c^ |  |  |  |
| Ln MEHP | -1.017 | -2.203, 0.169 | 0.092 |
| Ln maternal MEHP | -1.411 | -4.946, 2.124 | 0.432 |
| Model 6 ^b, c^ |  |  |  |
| Ln MEHHP | -1.253 | -2.647, 0.142 | 0.078 |
| Ln maternal MEHHP | -0.282 | -1.466, 0.902 | 0.639 |
| Model 7 ^b, c^ |  |  |  |
| **Ln MEOHP** | **-1.863** | **-3.116, -0.611** | **0.004** |
| Ln maternal MEOHP | 0.272 | -0.939, 1.484 | 0.658 |
| Model 8 ^b, c^ |  |  |  |
| **Ln ΣMEHP^d^** | **-1.587** | **-3.052, -0.122** | **0.034** |
| Ln maternal ΣMEHP^d^ | 0.153 | -2.213, 2.519 | 0.899 |
|  |  |  |  |

^a^The number of observations (n) represents the sum of all subjects studied at both birth and 1^st^ follow-up visit at 2 years of age, and at least once at 5, 8, or 11 year follow-up.

^b^adjusted for gender, HOME score, birth weight, maternal education, lactation, children’s age, children’s urinary creatinine levels, and maternal urinary creatinine levels.

^c^Maternal and children’s levels of urinary phthalate were both independent variables to predict IQ scores in the model.

^d^ΣMEHP= MEHP+ MEHHP+MEOHP.
