# Supplementary material for: Fetal and Childhood Exposure to Phthalate Diesters and Cognitive Function in Children Up to 12 Years of Age: Taiwanese Maternal and Infant Cohort Study
Source: PLoS One. 2015 Jun 29;10(6):e0131910. doi: 10.1371/journal.pone.0131910 (PMC4488303; doi:10.1371/journal.pone.0131910)
Supplement: S5 Table — (DOCX) [file pone.0131910.s006.docx]

**S5 Table.** Associations between intelligence quotient (IQ) scores and mothers’ and childrenʼs urinary phthalate concentrations by linear mixed model (n ^a^ =196), with excluding mother’s and children’s urinary creatinine below 2 g/L

| Variables (µg/g creatinine) | Beta | 95% CI | *p*-value |
| --- | --- | --- | --- |
| Model 1 ^b, c^ |  |  |  |
| Ln MMP | -0.991 | -2.226, 0.244 | 0.115 |
| Ln maternal MMP | -1.181 | -3.455, 1.092 | 0.306 |
| Model 2 ^b, c^ |  |  |  |
| Ln MEP | -0.573 | -1.626, 0.481 | 0.284 |
| Ln maternal MEP | 1.718 | -0.557, 3.993 | 0.137 |
| Model 3 ^b, c^ |  |  |  |
| Ln MBP | -0.787 | -2.893, 1.319 | 0.461 |
| Ln maternal MBP | 0.0202 | -2.318, 2.358 | 0.986 |
| Model 4 ^b, c^ |  |  |  |
| Ln MBzP | -0.916 | -2.242, 0.410 | 0.174 |
| Ln maternal MBzP | -0.235 | -3.733, 3.264 | 0.895 |
| Model 5 ^b, c^ |  |  |  |
| Ln MEHP | -1.049 | -2.370, 0.273 | 0.119 |
| Ln maternal MEHP | -1.229 | -3.732, 1.274 | 0.333 |
| Model 6 ^b, c^ |  |  |  |
| Ln MEHHP | -1.383 | -2.957, 0.190 | 0.084 |
| Ln maternal MEHHP | -0.0426 | -1.298, 1.213 | 0.947 |
| Model 7 ^b, c^ |  |  |  |
| Ln MEOHP | -1.355 | -2.934, 0.225 | 0.092 |
| Ln maternal MEOHP | 0.464 | -0.890, 1.818 | 0.499 |
| Model 8 ^b, c^ |  |  |  |
| Ln ΣMEHP^d^ | -1.522 | -3.188, 0.145 | 0.073 |
| Ln maternal ΣMEHP^d^ | 0.188 | -2.163, 2.538 | 0.875 |
|  |  |  |  |

^a^The number of observations (n) represents the sum of all subjects studied at both birth and 1^st^ follow-up visit at 2 years of age, and at least once at 5, 8, or 11 year follow-up.

^b^adjusted for gender, HOME score, birth weight, maternal education, lactation, and children’s age.

^c^Maternal and children’s levels of urinary phthalate were both independent variables to predict IQ scores in the model.

^d^ΣMEHP= MEHP+ MEHHP+MEOHP.
